# Pulsed Laser Porosification of Silicon Thin Films

**DOI:** 10.3390/ma9070509

**Published:** 2016-06-24

**Authors:** Christian Sämann, Jürgen R. Köhler, Morris Dahlinger, Markus B. Schubert, Jürgen H. Werner

**Affiliations:** Institute for Photovoltaics and Research Center SCoPE, University of Stuttgart, Pfaffenwaldring 47, 70569 Stuttgart, Germany; juergen.koehler@ipv.uni-stuttgart.de (J.R.K.); morris.dahlinger@ipv.uni-stuttgart.de (M.D.); markus.schubert@ipv.uni-stuttgart.de (M.B.S.); juergen.werner@ipv.uni-stuttgart.de (J.H.W.)

**Keywords:** pulsed laser, porous, silicon, thin film, gas bubbles

## Abstract

We present a new and simple laser-based process to porosify thin film silicon using a pulsed laser. During deposition, we incorporate gas atoms or molecules into the Si thin film. Pulsed laser radiation of wavelength λ=532nm heats up thin film Si beyond its melting point. Upon heating, gas atoms or molecules form nm-sized thermally expanding gas bubbles in the silicon melt, until they explosively exit the film, leaving pores behind. Rapid heating and fast cooling during pulsed laser processing enable re-solidification of the liquid Si before the created pores contract and pore closure occurs within the liquid phase. Optimized plasma-enhanced chemical vapor deposition or sputtering of amorphous Si thin films on stainless steel substrate incorporates the necessary concentration of gas atoms or molecules. We are able to tailor the pore size between 50 and 550 nm by changing laser pulse energy density and film deposition parameters. Evaporated silicon containing no gas atoms forms only a few very large μm-sized gas bubbles due to laser-induced vapor formation of evaporated solid material at the substrate–silicon interface.

## 1. Introduction

The research on porous silicon (Si) is of current interest due to its various applications [1]. Sensors use the high surface area-to-volume ratio of porous Si to increase their sensitivity in chemical analysis. Schechter et al. used the increase of several orders of magnitude in electrical conductivity due to vapor pressure variations for gas sensing [2]. The voids in porous silicon also deteriorate the thermal conductivity of porous silicon; therefore, a potential application is thermoelectric energy conversion [3]. We use porous Si in lithium (Li)-ion batteries as anode material in high energy density batteries. Si expands up to 270% during lithium insertion, which causes mechanical fracturing [4]. Therefore, the porosity increases its cyclic stability, because the voids provide space for volume expansions.

Porous Si is usually produced by wet chemical etching of crystalline Si wafers. The most common method and well established process is anodic etching [5,6]. A Si wafer immersed in aqueous hydrofluoric acid (HF) and biased connected to a positive electrode creates homogeneous porous structures. The dissolution of Si produces well-defined pores several microns deep in a diameter range between nanometers and micrometers. Another variation of the wet chemical etching facilitates the etching in HF and H_2_O_2_ by a less than 10 nanometers thin metal layer on the surface. Depending on the metal and the doping of Si, different porosities using different etching rates are produced due to local redox reactions [7]. The stain etching method uses HF and small amounts of a chemical oxidant (HNO_3_ or CrO_3_) to produce porous films on top of the Si wafer. The thickness of the porous films produced by stain etching is limited to 100 nm in thickness [8]. Bao et al. presented a process to produce microporous silicon by magnesiothermic reduction of silica [9], which Chen et al. applied for mesoporous silicon anodes for Li-ion batteries [10].

The present contribution presents a new method using pulsed laser radiation to porosify Si thin films. Successful porosification requires incorporated gas atoms within the Si film. We show that hydrogen in plasma enhanced chemical vapor deposited (PECVD) Si and Argon (Ar) in sputtered Si fulfill this requirement and allow the porosification with pulsed laser radiation. The gas atoms serve as a source for explosively expanding gas bubbles to create porous thin film Si.

## 2. Experimental Section

### 2.1. Laser Porosification Process

**Figure 1:**
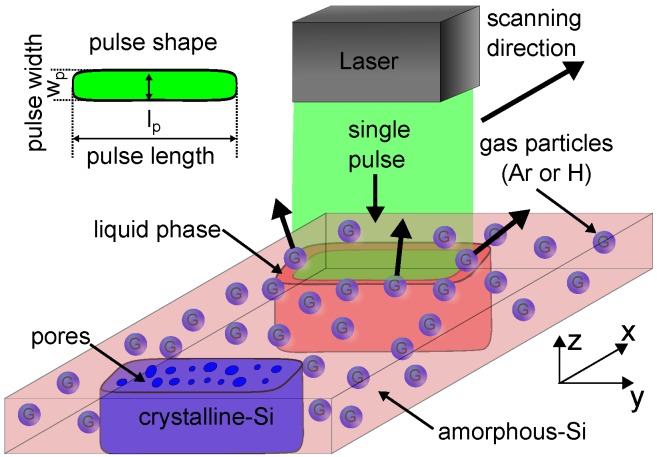
Laser process for porous thin film Si illustrates our theory for pore creation. Amorphous thin film Si absorbs the energy of line shaped single laser pulses with pulse width $w_{p}=3\;\mu$m and pulse length $l_{p}=800\;\mu m$. The temperature of the amorphous Si heats up until a phase change from solid to liquid occurs. Incorporated gas particles/molecules accumulate to thermal expanding bubbles until they explosively exit the film, leaving pores behind. A fast cool down due to pulse durations between 100 ns and 200 ns and solidification creates multi-crystalline porous thin film Si.

Figure 1 schematically shows the laser process to produce porous thin film Si. We use a frequency doubled Nd:YAG laser emitting pulsed laser radiation of wavelength λ=532nm. A purpose-designed optics shapes the circular laser beam to a top-hat shaped line focus at the focal plane of the optics. The aspect ratio of the line focus is *a* = $l_{p}$/$w_{p}$ = 267, with $l_{p}=800\;\mu$m the long axis in the y-direction and $w_{p}=3\;\mu$m the short axis in the x-direction of the line focus. The Gaussian energy distribution of the laser pulse along the short axis causes a maximum energy density in the center of the beam. We calculate the laser pulse energy density $H_{P}=Q_{P}/4\sigma l_{p}$, using the area where the beam intensity drops to $1/e^{2}$. $Q_{P}$ is the laser pulse energy and *σ* is the standard deviation. Amorphous Si deposited on stainless steel substrate is fixed to an x/y translation stage. The stage moves the substrate in direction *x* with a constant velocity $v=150\;{\mathrm{mms}}^{-1}$, which is equivalent to scanning the laser beam over the substrate in direction *x*. A laser pulse repetition frequency f=20kHz results in several microns of space between two subsequent laser pulses. The comparison of single laser pulses is necessary to understand the details of the laser process. Our theory for the mechanism of pore creation assumes that during irradiation, the amorphous Si thin film absorbs the laser radiation of the single pulses and heats up beyond the melting point. The presence of gas atoms or molecules within the Si film is indispensable for porosification. After a phase change from solid to liquid, the incorporated gas atoms or molecules within the Si film become mobile and accumulate to gas bubbles, which further expand due to temperature rise until they explosively exit the film, leaving pores behind. Due to laser pulse durations in the range between 100 and 200 ns, the liquid silicon re-solidifies before the created pores contract and pore closure occurs within the liquid phase. The pore size distribution depends both on the fluence as well as on the gas type and its concentration within the film.

### 2.2. Thin Film Silicon on Stainless Steel Foil Substrates

The rolled stainless steel (X5CrNi18-10) foil substrates of thickness $d_{\mathrm{SF}}=25\;\mu$m are cleaned before deposition in two steps—first by acetone, followed by isopropyl alcohol to remove organic contaminants and improve the film adhesion. The Si thin film thickness $d_{\mathrm{Si}}=200\;\mathrm{nm}$ is equal for all used deposition methods in order to have similar absorption of the pulsed laser radiation.

#### 2.2.1. Electron Beam Evaporated Films

As a reference, we use evaporated Si for thin film Si containing no gas atoms. Evacuation of the recipient to base pressure $p=2\times 10^{-7}\;\mathrm{mbar}$ before deposition prevents significant gas incorporation in Si thin films. The deposition system uses an electron beam to melt and evaporate Si out of a crucible. Laser porosification of these films is not possible, since the required gas atoms or molecules are not incorporated into the Si film.

#### 2.2.2. Sputtered Films

Winters and Kay showed the relation between sputter pressure and argon (Ar) concentration in sputtered nickel films [11]. They measured a decreasing Ar content in the thin film with increasing sputtering pressure using laser-induced flash evaporation and mass spectrometry. Pawlewicz determined the Ar content in sputtered amorphous Si thin films, confirming the results of Winters and Kay using X-ray energy spectrometry [12]. Chapman attributes the incorporation of Ar to Ar ions that are neutralized after hitting the target and reaching the substrate. The high energetic neutrals are likely to be incorporated into the thin films. The energy of neutrals reaching the surface decreases with the pressure due to their increased collision frequency in the deposition chamber. An increase of the pressure during deposition therefore results in a decrease of the kinetic energy of reflected neutrals, thus less Ar is incorporated [13].

We deposit sputtered Si layers with a Leybold Z 550 radio-frequency (RF) sputtering system operating at a frequency f=13.56MHz using a crystalline Si target. The background pressure *p* of the sputter recipient is in the range of $p=5\times 10^{-5}\;\mathrm{mbar}$. The sputtering pressure during deposition is $p=6\times 10^{-3}\;\mathrm{mbar}$. The deposition at room temperature results in an amorphous growth of Si on the stainless steel substrates and incorporation of Ar from the sputter gas. For porosification, we incorporate Ar, which prevents chemical bondings due to its inert property.

### 2.3. PECVD Films

Plasma enhanced chemical vapor deposited thin film Si contains chemically bonded hydrogen, which is usually used to saturate dangling bonds in amorphous Si to improve electrical properties of the thin film. During deposition, the plasma containing Ar^+^ ions dissociate SiH_4_ molecules, resulting in thin film Si growth. Knights showed the relation of decreasing hydrogen content with increasing substrate temperature during deposition [14]. Substrate temperature $T_{\mathrm{sub}}=170{\;}^{\circ }$C and deposition pressure p=0.5mbar during deposition gives an amorphous growth of silicon on the cleaned stainless steel foil substrates. Gas flow F=3sccm silane plus F=3sccm phosphine (2% solved in silane) and RF power P=2W deposits hydrogenated amorphous Si. We use the hydrogen that is chemically bonded to Si for porosification. In contrast to the inert Ar within the sputtered Si film, the reactivity of hydrogen might influence the porosification process.

**Figure 2:**
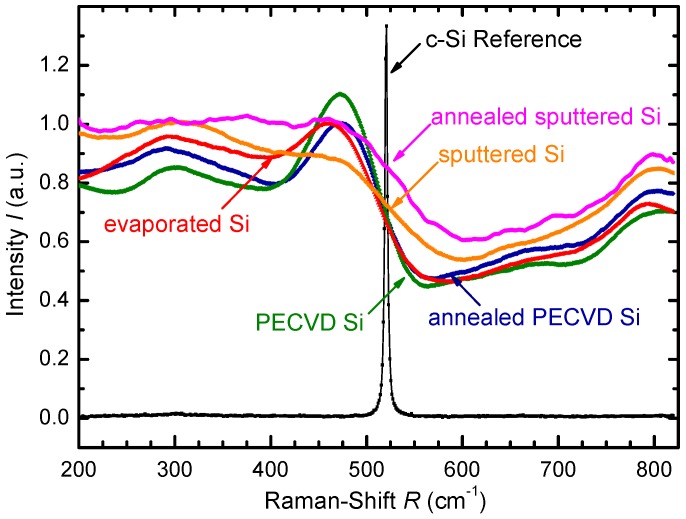
Raman spectra of different thin film Si deposited on $d_{\mathrm{SF}}=25\;\mu m$ thick stainless steel foil substrates. The sharp peak at Raman-Shift $R=520\;{\mathrm{cm}}^{-1}$ stems from the characteristic crystalline Si reference. The spectra of evaporated, sputtered and PECVD Si thin films indicate amorphous film growth. PECVD and sputtered Si show no crystalline peak after tempering at $T=350{\;}^{\circ }$C for t=15 min.

Figure 2 shows the results of the Raman spectroscopy of the Si samples deposited on stainless steel foil substrates. The excitation source for Raman spectroscopy was a green laser with wavelength λ=532nm. The reference peak at Raman-Shift $R=520\;{\mathrm{cm}}^{-1}$ is characteristic for crystalline Si. The spectra of sputtered, PECVD, and evaporated Si samples prove the amorphous film growth of all three deposition methods. PECVD and sputtered Si stayed amorphous after annealing at $T=350{\;}^{\circ }$C for t=15 min on a hot plate.

## 3. Results and Discussion

### 3.1. Raman Spectroscopy

The Raman spectra of the thin films for different fluence $H_{P}$ are presented in order to show the amorphous growth and the laser-induced crystallization of the Si thin films. For stage velocities in the x direction down to $v=65\;{\mathrm{mms}}^{-1}$, the subsequent laser pulses overlap; hence, full area laser irradiation of the samples is achieved.

**Figure 3:**
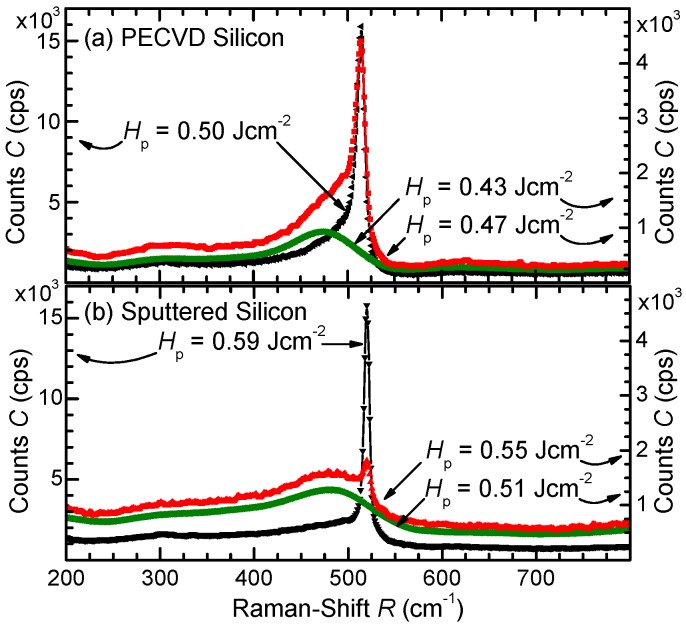
Raman spectra of pulsed laser irradiated thin film Si. The thickness $d_{\mathrm{Si}}$ of all Si films is 200 nm. (**a**) PECVD Si is amorphous after irradiation with fluence $H_{P}=0.43\;{\mathrm{Jcm}}^{-2}$ and starts crystallizing for fluence $H_{P}=0.47\;{\mathrm{Jcm}}^{-2}$. Rising the fluence $H_{P}=0.50\;{\mathrm{Jcm}}^{-2}$ increases the intensity of the crystalline Si peak at Raman-Shift $R=520\;{\mathrm{cm}}^{-1}$; (**b**) Sputtered Si is amorphous after irradiation with fluence $H_{P}=0.51\;{\mathrm{Jcm}}^{-2}$ and starts to crystallize at fluence $H_{P}=0.55\;{\mathrm{Jcm}}^{-2}$, and fluence $H_{P}=0.59\;{\mathrm{Jcm}}^{-2}$ increases crystalline Si peak intensity.

Figure 3a,b show the results of Raman spectroscopy of PECVD and sputtered samples respectively, comparing the crystallization threshold fluence. Si layer thickness is $d_{\mathrm{Si}}=200\;\mathrm{nm}$ for all samples deposited on $d_{\mathrm{SF}}=25\;\mu$m thick stainless steel foil substrates. The exciting source is a line focused green laser with wavelength λ=532nm in an inVia Raman microscope by *Renishaw plc*. Figure 3a shows the Raman spectra of laser processed PECVD Si films, which are still amorphous after irradiation with fluence $H_{P}=0.43\;{\mathrm{Jcm}}^{-2}$. The main peak of the Raman spectrum shifts to the characteristic transverse optic Raman peak of single crystalline Si at $R=520\;{\mathrm{cm}}^{-1}$ after increasing the fluence to $H_{P}=0.47\;{\mathrm{Jcm}}^{-2}$. Irradiation with $H_{P}=0.50\;{\mathrm{Jcm}}^{-2}$ increases the intensity at the wave number of the crystalline Si peak and narrows the peak itself, resulting from further laser induced crystallization. Figure 3b shows Raman spectra for sputtered Si, which are still amorphous after irradiation up to a fluence of $H_{P}=0.51\;{\mathrm{Jcm}}^{-2}$, and shows only a small crystalline Si peak after irradiation with $H_{P}=0.55\;{\mathrm{Jcm}}^{-2}$. Irradiation with fluence $H_{P}=0.59\;{\mathrm{Jcm}}^{-2}$ completely crystallizes the sputtered Si, indicated by the sharp peak.

### 3.2. Scanning Electron Microscopy

#### 3.2.1. Evaporated Silicon

**Figure 4:**
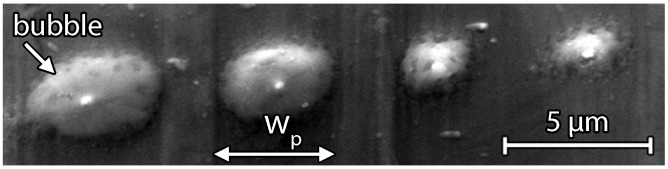
SEM image of laser treated electron beam evaporated $d_{\mathrm{Si}}=200\;\mathrm{nm}$ thick Si on stainless steel foil. Fluence $H_{P}=0.51\;{\mathrm{Jcm}}^{-2}$ causes Si bubbles without pore formation. Underlying substrate structure might be the reason for the bubble formation along a line within the pulse width $w_{p}$ of different laser pulses.

Evaporation of silicon in vacuum with a base pressure $p=2\times 10^{-7}\;\mathrm{mbar}$ before deposition prevents significant gas incorporation in Si thin films. Figure 4 shows a scanning electron microscopy (SEM) image of $d_{\mathrm{Si}}=200\;\mathrm{nm}$ thick evaporated thin film silicon after laser irradiation with a fluence $H_{P}=0.51\;{\mathrm{Jcm}}^{-2}$. As evaporated silicon contains no gas atoms, only a few very large μm-sized gas bubbles due to laser induced vapor formation of evaporated solid material at the substrate–silicon interface are formed [15]. The four visible large-sized bubbles are formed in four single laser pulse treated areas. Each area shows a silicon bubble, which occurs along the line focused laser beam. The stainless steel foil substrate has a textured surface due to line-shaped scratches from the rolling process during production. Since all bubbles occur along a line in each single pulse, the underlying substrate scratches might be the reason for the bubble formation. We believe that material from the stainless steel substrate evaporates preferably in those scratches, leading to the formation of the μm-sized bubbles. Increasing the fluence cracks the Si bubbles and results in holes through the entire Si film, which makes evaporated silicon useless for laser porosification.

#### 3.2.2. PECVD Silicon

**Figure 5:**
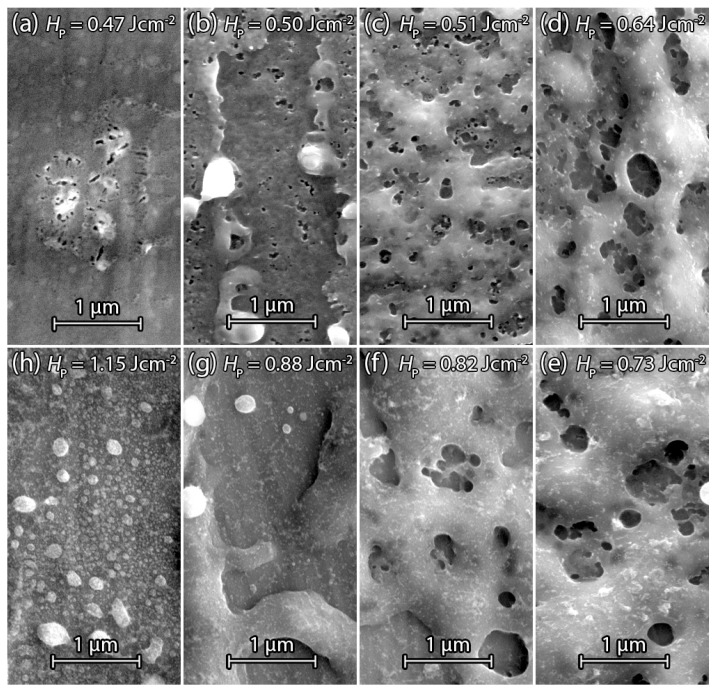
SEM surface images of laser treated PECVD Si on stainless steel foil. No annealing before laser irradiation. (**a**) Surface of Si treated by the threshold fluence $H_{P}=0.47\;{\mathrm{Jcm}}^{-2}$ for observable pore formation; The increase in irradiation with fluence $H_{P}=0.50\;{\mathrm{Jcm}}^{-2}$ in (**b**) to $H_{P}=0.51\;{\mathrm{Jcm}}^{-2}$ in (**c**) results in enhanced pore formation; Pore formation in (**c**) occurs on the whole surface of the irradiated area; (**d**) Further fluence increase to $H_{P}=0.64\;{\mathrm{Jcm}}^{-2}$ and $H_{P}=0.73\;{\mathrm{Jcm}}^{-2}$ in (**e**) increase the pore diameter; (**f**) Fluence $H_{P}=0.82\;{\mathrm{Jcm}}^{-2}$ keeps Si liquid for long enough to close most of the pores fully or partially; (**g**) A single pulse with fluence $H_{P}=0.82\;{\mathrm{Jcm}}^{-2}$ displaced Si from the pulse center to the boundary without any noticeable pore formation; (**h**) Fluence $H_{P}=1.51\;{\mathrm{Jcm}}^{-2}$ heats Si layer over the boiling point. Droplets condense in the pulse center.

Figure 5a–h present scanning electron microscopy (SEM) detail images of laser irradiated $d_{\mathrm{Si}}=200\;\mathrm{nm}$ thick PECVD Si films in the center of the laser pulse. Figure 5a shows the Si surface after the irradiation with fluence $H_{P}=0.47\;{\mathrm{Jcm}}^{-2}$, which is the threshold energy density for observable pore formation. Very small pores of average diameter $d_{P}=100\;\mathrm{nm}$ are only visible in the center of the laser pulse. An increase of fluence to $H_{P}=0.50\;{\mathrm{Jcm}}^{-2}$ in Figure 5b creates a higher pore density at the center of the laser pulse. Pores still only exist close to the surface. Figure 5c shows a pore distribution over the whole detail image. In comparison to Figure 5a,b, the whole surface area undergoes the phase change from solid amorphous to liquid and back to crystalline solid Si. Figure 5d shows that a further increase of the fluence to $H_{P}=0.64\;{\mathrm{Jcm}}^{-2}$ increases the average pore size in the center of the pulse. The melting depth is approximately proportional to the fluence and hence the amount of hydrogen molecules involved in the process increases [16]. Therefore, the average gas bubble size—and finally, the pore diameter—increases. Figure 5e shows a PECVD sample irradiated with a fluence of $H_{P}=0.73\;{\mathrm{Jcm}}^{-2}$, revealing a lower porosity and average pore diameter at the surface compared to the surfaces in Figure 5c,d. Not only the melting depth but also the duration for which Si stays liquid before it re-solidifies increases when fluence increases [17]. If Si stays liquid for too long after the escape of gas bubbles, the created pores start to close. Figure 5f shows that a further increase of the fluence to $H_{P}=0.82\;{\mathrm{Jcm}}^{-2}$ enhances pore closure, resulting in a decreasing number of pores and reduced average pore diameter. Figure 5g shows the center of the irradiated area using a fluence of $H_{P}=0.88\;{\mathrm{Jcm}}^{-2}$. Most of the Si is transported from the center to the boundaries of the irradiated area, probably due to temperatures reaching the boiling point of Si. Evaporated Si leads to a significantly high gas pressure above the center zone, which pushes the Si aside as long as it is liquid. In the very center of the pulse, one can see re-condensed droplets from evaporated Si. Further increase of the fluence to $H_{P}=1.15\;{\mathrm{Jcm}}^{-2}$ as depicted in Figure 5h evaporates the irradiated Si completely. The evaporated Si re-condenses and covers the center with small droplets.

#### 3.2.3. Annealed PECVD Silicon

Annealing of the $d_{\mathrm{Si}}=200\;\mathrm{nm}$-thick PECVD Si samples on stainless steel foils at temperature $T=350{\;}^{\circ }$C for the time t=15 min on a hot plate reduces the amount of incorporated hydrogen by effusion [18]. A comparison of annealed PECVD laser irradiated samples with non-annealed samples in Figure 5a–h shows the influence of hydrogen reduction for different fluences.

**Figure 6:**
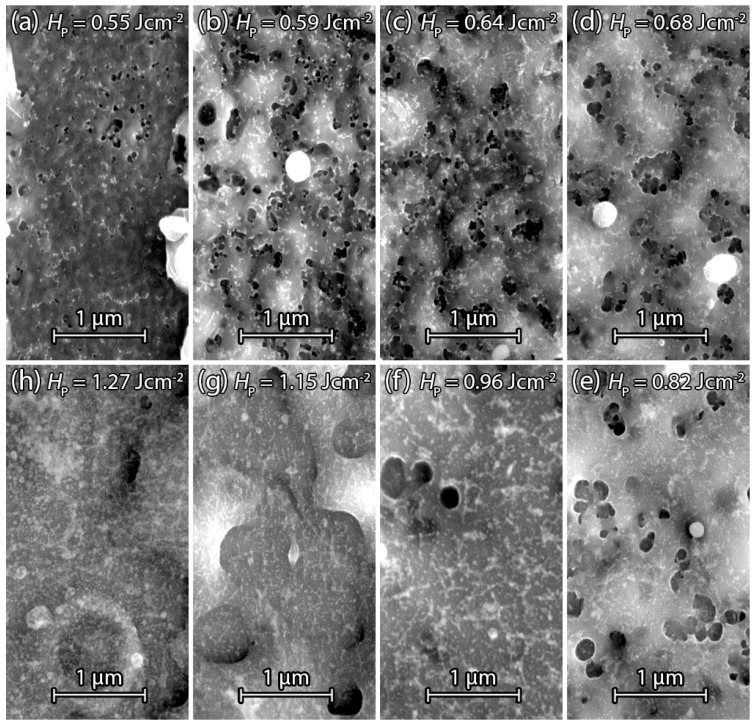
SEM images of laser treated PECVD Si on stainless steel foil prior annealed for t=15 min at $T=350{\;}^{\circ }$C. (**a**) Surface image of Si irradiated by a fluence $H_{P}=0.55\;{\mathrm{Jcm}}^{-2}$ single pulse showing the threshold for pore formation; (**b**) Fluence $H_{P}=0.59\;{\mathrm{Jcm}}^{-2}$ causes an increase of pores formation; The gradually increasing fluence $H_{P}=0.64\;{\mathrm{Jcm}}^{-2}$ in (**c**) and fluence $H_{P}=0.68\;{\mathrm{Jcm}}^{-2}$ in (**d**) cause the enlargement of the formed pores; (**e**) Further increase of fluence $H_{P}=0.82\;{\mathrm{Jcm}}^{-2}$ reduces the number of pores and pore diameter; (**f**) Fluence $H_{P}=0.96\;{\mathrm{Jcm}}^{-2}$ keeps Si liquid long enough so that pores are mostly closed again; (**g**) Further fluence increase to $H_{P}=1.15\;{\mathrm{Jcm}}^{-2}$ displaces Si from the pulse center to the boundaries; (**h**) Fluence $H_{P}=1.27\;{\mathrm{Jcm}}^{-2}$ leaves only condensed Si on the surface of pulse center.

Figure 6a–h present SEM surface images of annealed PECVD Si samples after the laser treatment. Figure 6a shows the Si surface after irradiation with the threshold fluence $H_{P}=0.55\;{\mathrm{Jcm}}^{-2}$. The annealing step causes an increase of the threshold fluence compared to Figure 5a without annealing. Further increase of fluence from $H_{P}=0.59\;{\mathrm{Jcm}}^{-2}$ in Figure 6b up to $H_{P}=0.96\;{\mathrm{Jcm}}^{-2}$ in Figure 6f shows the same behavior as the sample in Figure 5a–h. The pore diameter $d_{P}$ increases with increasing fluence $H_{P}$ until a certain point and starts to decrease with further fluence $H_{P}$ increase. The main difference is the shift to higher fluences due to hydrogen effusion by the annealing step. In Figure 6g the fluence $H_{P}=1.15\;{\mathrm{Jcm}}^{-2}$ results in a displacement of Si from the pulse center to non irradiated surrounding area. Further increase of fluence to $H_{P}=1.27\;{\mathrm{Jcm}}^{-2}$ in Figure 6h indicates re-condensed Si after boiling or evaporation similar to Figure 5h.

**Figure 7:**
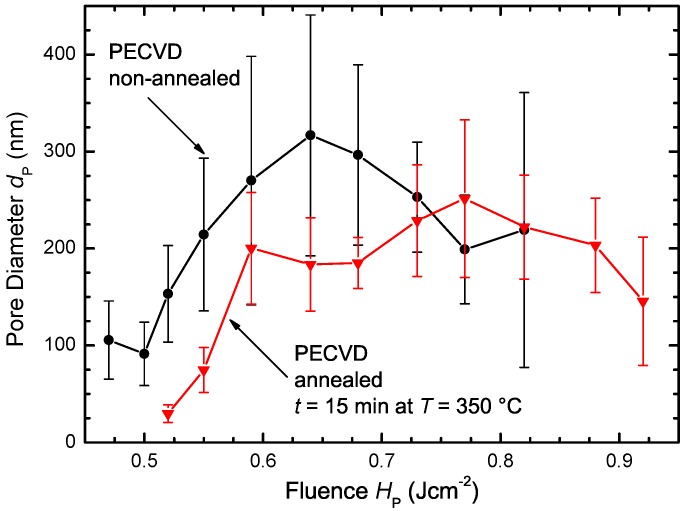
Average pore diameter $d_{P}$ with standard deviation in dependence of the fluence $H_{P}$. The ten largest pore diameters $d_{P}$ were measured by hand for each fluence $H_{P}$. Pore diameter $d_{P}$ increases and decreases after reaching maximum. The pore diameters $d_{P}$ of the annealed PECVD samples are shifted to higher fluences $H_{P}$ due to hydrogen effusion.

Figure 7 shows the average pore diameter $d_{P}$ with standard deviation in dependence of fluence $H_{P}$ for annealed and non-annealed PECVD samples. For each fluence $H_{P}$, the ten largest pore diameters within the detail SEM images were measured via visual inspection. When fluence $H_{P}$ exceeds the threshold fluence $H_{\mathrm{Pth}}$ for observable pore formation, the pore diameter $d_{P}$ increases with increasing fluence $H_{P}$ until the melt duration of Si is long enough for pore closure. Hydrogen effusion in annealed PECVD samples for t=15 min at $T=350{\;}^{\circ }$C before laser treatment results in a fluence shift to higher energy densities to obtain similar pore diameters $d_{P}$. Higher fluences compensate for the hydrogen loss to some extent via deeper melting to involve more hydrogen in the gas bubble formation. The lower content of hydrogen also results in a decrease of the maximum average pore diameter $d_{P}$ for the annealed PECVD samples.

#### 3.2.4. Sputtered Silicon

**Figure 8:**
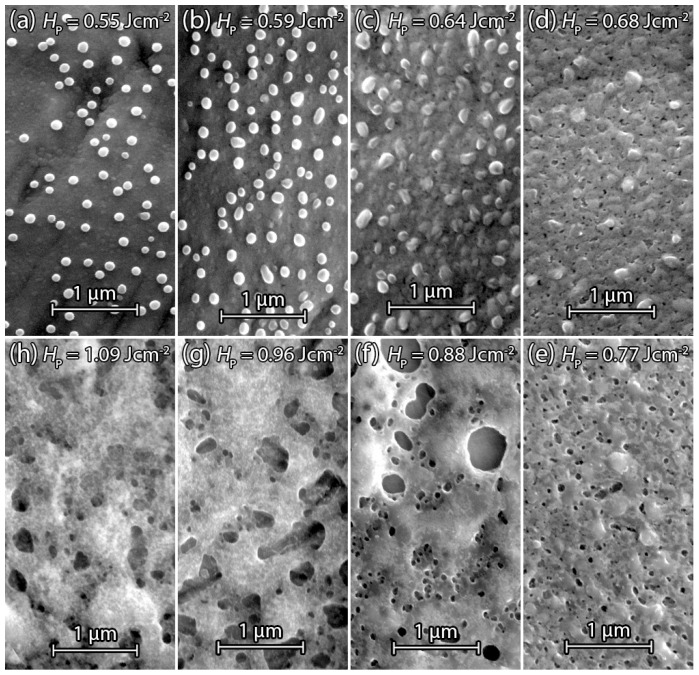
SEM surface images of sputtered $d_{\mathrm{Si}}=200\;\mathrm{nm}$ thick Si on $d_{\mathrm{SF}}=25\;\mu$m stainless steel foil after laser irradiation. (**a**) Irradiation with fluence $H_{P}=0.55\;{\mathrm{Jcm}}^{-2}$ causes pellet formation on the surface due to local liquefied silicon, which coagulates during cool-down; (**b**) Increase of fluence to $H_{P}=0.59\;{\mathrm{Jcm}}^{-2}$ enhances pellet formation; (**c**) Pellets got melted into the Si surface by fluence $H_{P}=0.64\;{\mathrm{Jcm}}^{-2}$; (**d**) Irradiation by fluence $H_{P}=0.68\;{\mathrm{Jcm}}^{-2}$ cause first pore formation between the melted pellets; (**e**) Increase of fluence to $H_{P}=0.77\;{\mathrm{Jcm}}^{-2}$ increases the amount of pores and pore diameter; (**f**) Fluence $H_{P}=0.88\;{\mathrm{Jcm}}^{-2}$ melts the Si surface and creates pore diameters of several hundred nanometers; (**g**) Rough surface and decreasing pore diameter despite increasing fluence $H_{P}=0.96\;{\mathrm{Jcm}}^{-2}$, due to evaporation and condensing Si; (**h**) Further reduction of pore size due to irradiation with fluence $H_{P}=1.09\;{\mathrm{Jcm}}^{-2}$ by filling pores with condensed Si.

Figure 8a–h show SEM surface images of laser irradiated sputtered Si on stainless steel foil $d_{\mathrm{SF}}=25\;\mu$m. The same Si film thickness $d_{\mathrm{Si}}=200\;\mathrm{nm}$ allows a comparison with laser treated PECVD Si samples. Figure 8a shows the center of a single laser pulse with the fluence $H_{P}=0.55\;{\mathrm{Jcm}}^{-2}$ treated Si surface. In contrast to the PECVD samples, the surface of the laser irradiated area is covered with small spherically-shaped droplet-like Si particles. A possible explanation for droplet formation is that the surface is not made entirely molten by the laser pulse, but the temperature reaches a significantly high value so that a phase transition between amorphous and crystalline silicon via explosive crystallization (EC) can occur. Here, the amorphous phase has a higher free energy than the crystalline phase and will therefore tend to crystallize under the release of latent heat [19]. Explosive crystallization therefore can lead to a partial crystallization of Si, starting from “hot spots” at the surface, which subsequently consume the surrounding amorphous silicon. After EC, the temperature of the crystallized areas is hotter than the surrounding amorphous material, and further laser irradiation leads to liquid islands, which coagulate due to the high surface tension, and form droplets on the surface. Figure 8b shows the Si surface after irradiation with a fluence of $H_{P}=0.59\;{\mathrm{Jcm}}^{-2}$. The increase of fluence results in augmented droplet formation. Figure 8c shows the laser treated Si surface after a further increase of the fluence to $H_{P}=0.64\;{\mathrm{Jcm}}^{-2}$. Here the surrounding liquid silicon remelts the droplets, which indicates a fully liquid Si surface which reduced the coagulation of the liquid Si during its cool down. Figure 8d shows the beginning of pore formation. A fluence of $H_{P}=0.68\;{\mathrm{Jcm}}^{-2}$ was sufficient to heat Si long and deep enough above its melting point to activate Ar atoms for gas bubble formation. Droplets are still present at the surface but have fewer irregularities compared to Figure 8a–c. Figure 8e shows a significant formation of pores. The fluence of $H_{P}=0.77\;{\mathrm{Jcm}}^{-2}$ enhances pore formation by a deeper melting, also resulting in an almost complete extinction of the droplets. Figure 8f shows a broadened pore size distribution caused by the increased fluence of $H_{P}=0.88\;{\mathrm{Jcm}}^{-2}$. The Si surface was completely molten and solidifies homogeneously between pores. The surrounding surface area of large pores shows a significant reduction of small pores, which indicates the consumption of surrounding incorporated Ar for large pore formation. Sputtered Si irradiated with fluence $H_{P}=0.96\;{\mathrm{Jcm}}^{-2}$ in Figure 8g shows a change in surface morphology compared to surfaces that have been irradiated with lower fluence. An explanation for the change in morphology might be a local heating of Si beyond the boiling point, causing evaporation followed by a re-condensation on the surface. Further increase of fluence to $H_{P}=1.09\;{\mathrm{Jcm}}^{-2}$ in Figure 8h shows a similar surface to Figure 8g, except that the pores get more and more covered or filled by the evaporated and condensed Si.

#### 3.2.5. Annealed Sputtered Silicon

**Figure 9:**
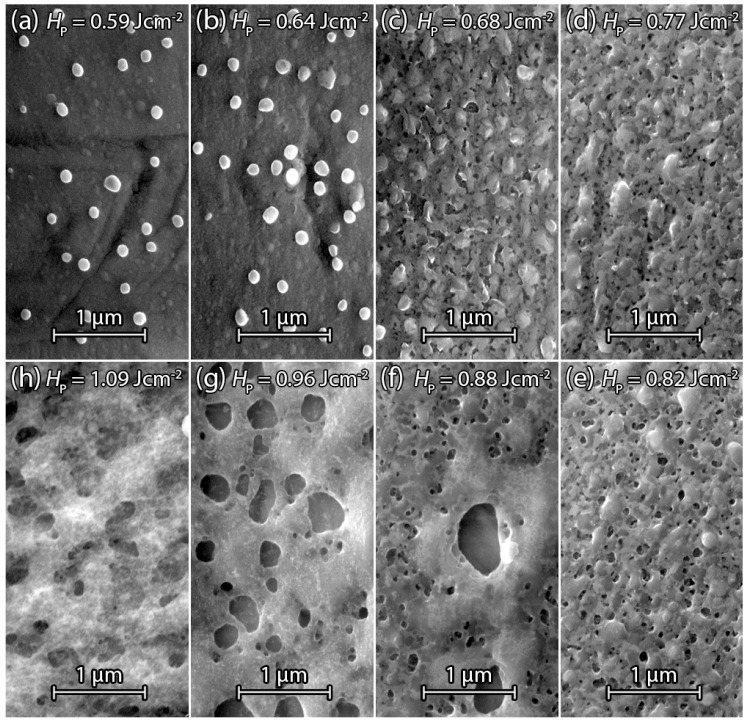
SEM surface images of sputtered Si on stainless steel foil annealed for t=15 min at T=350${}^{\circ }$C before laser irradiation. (**a**) Fluence $H_{P}=0.59\;{\mathrm{Jcm}}^{-2}$ causes less pellets on the surface compared to the same fluence-treated surface in Figure 8b; (**b**) Increase of fluence to $H_{P}=0.64\;{\mathrm{Jcm}}^{-2}$ causes a slight increase in pellet formation; (**c**) Further fluence increase to $H_{P}=0.68\;{\mathrm{Jcm}}^{-2}$ melts pellets and first pore formation takes place; (**d**) Gain of pore formation within the same pore diameter range as in (**c**) by irradiation with fluence $H_{P}=0.73\;{\mathrm{Jcm}}^{-2}$; (**e**) Pore diameter size increases by raising the fluence to $H_{P}=0.82\;{\mathrm{Jcm}}^{-2}$; (**f**) Fluence $H_{P}=0.88\;{\mathrm{Jcm}}^{-2}$ causes a single pore in the center that consumed the surrounding Ar indicated by almost no pores at the surrounding surface; (**g**) Pore size diameter increased at the fluence $H_{P}=0.96\;{\mathrm{Jcm}}^{-2}$. Pore surrounding Si surface is free of pores; (**h**) Si surface gets rough after laser irradiation with fluence $H_{P}=1.09\;{\mathrm{Jcm}}^{-2}$ and pore size diameters decrease.

In contrast to annealed PECVD samples, tempering of sputtered Si for t=15min at T=350 °C on a hot plate before laser irradiation shows no significant change—neither in the threshold fluence $H_{\mathrm{Pth}}$ for observable pore formation, nor in the average pore diameter $d_{P}$. Figure 9a–h show SEM surface images of sputtered Si on stainless steel foil after tempering and laser processing. Figure 9a,b with fluence $H_{P}=0.59\;{\mathrm{Jcm}}^{-2}$, $H_{P}=0.64\;{\mathrm{Jcm}}^{-2}$ show no pore formation similar to the samples in Figure 8a–c. Pore formation starts at a fluence of $H_{\mathrm{Pth}}$, like in the un-tempered samples. Compared to the sample in Figure 8b treated with the same fluence, the only difference is a slight reduction of the density of droplets on the Si surface and a slightly increased average droplet diameter. Whether a reduced Ar content in the film is responsible for this difference is not yet clear. Figure 9c–h demonstrate that pore formation and re-melted droplets on the surface is comparable to the samples in Figure 8d–h, indicating that tempering does not affect pore formation significantly.

**Figure 10:**
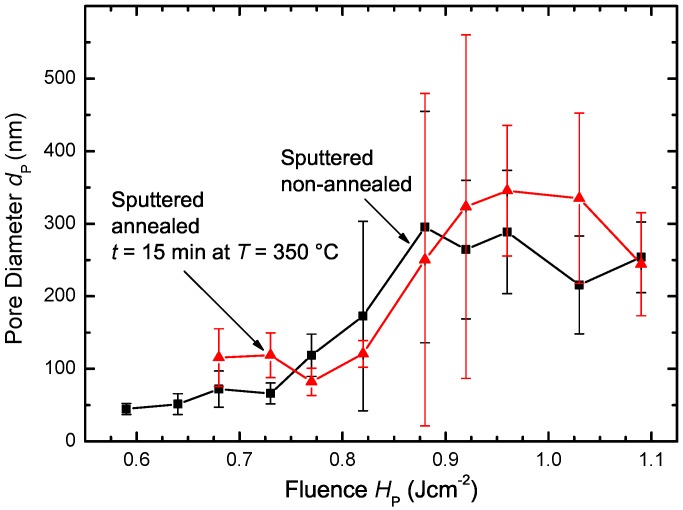
Pore diameter $d_{P}$ in dependence of fluence $H_{P}$ for sputtered non-annealed Si and annealed for t=15 min at T=350${}^{\circ }$C before laser irradiation. The ten largest pore diameters for each fluence $H_{P}$ were measured by hand. Tempered Si requires higher fluence $H_{P}$ for observable pore formation. There is no significant change in pore diameters $d_{P}$ caused by the tempering step for fluence $H_{P}>0.75\;{\mathrm{Jcm}}^{-2}$. A maximum in pore diameter $d_{p}$ exists for $0.9\;{\mathrm{Jcm}}^{-2}<H_{P}<1.05\;{\mathrm{Jcm}}^{-2}$, followed by a decrease in pore diameter $d_{P}$ due to liquid silicon causing the pores to start closing again.

Figure 10 shows the pore diameter $d_{P}$ of the non-annealed and annealed sputtered Si films in dependence of the applied fluence $H_{P}$. Even though the graphs for annealed and non-annealed Si films exhibit differences, the uncertainty for the data points indicated by the error bars is comparable to the differences of the measured pore diameter. Therefore, annealing does not significantly affect pore formation in sputtered Si. The reason is probably the larger atomic mass and diameter, making Ar at the thermal budget during the annealing step less mobile compared to hydrogen. The resulting lower diffusivity in amorphous Si keeps Ar within the film during annealing.

The comparison of the porosity of PECVD Si with sputtered Si shows a difference in the surface structure. Only sputtered Si shows pellet formation before pore formation occurs. Apparently the type of gas and its reactivity influence the porosification process. Nevertheless, both kinds of Si contain gas atoms and show an increase in pore diameter with increasing fluence. Evaporated Si containing no gas as reference shows no pore formation after laser irradiation and both PECVD Si and sputtered Si—which contain gas atoms—show pore formation, which supports our theory for the mechanism of pore formation.

## 4. Conclusions

We present a new method to produce porous Si thin films with pulsed laser radiation of wavelength λ=532nm. Successful porosification requires incorporated gas atoms, which must be inserted during deposition. Thin film Si with Ar sputtered Si and hydrogenated PECVD Si approved Ar and hydrogen as sources for thermal expanding gas bubbles, which create pores that remain after re-solidification of Si. The melting depth and the melting duration of silicon depends on the fluence $H_{P}$ and determines the amount of gas participating in the growth of thermally expanding bubbles. Therefore, we can adjust the pore diameter distribution of the thin film Si by varying the deposition parameters (which influence the content of incorporated gas particles) and by varying the fluence $H_{P}$.
